# Relationships between population traits, nonstructural carbohydrates, and elevation in alpine stands of *Vaccinium myrtillus*

**DOI:** 10.1002/ajb2.1458

**Published:** 2020-04-01

**Authors:** Valentino Casolo, Enrico Braidot, Elisa Petrussa, Marco Zancani, Angelo Vianello, Francesco Boscutti

**Affiliations:** ^1^ Department of Agriculture, Food, Environmental and Animal Sciences Plant Biology Unit University of Udine via delle Scienze 91 33100 Udine Italy

**Keywords:** bilberry, carbon stores, climate change, elevation stress, Ericaceae, functional traits, phenotypic integration, population traits, treeline acclimation

## Abstract

**Premise:**

Despite great attention given to the relationship between plant growth and carbon balance in alpine tree species, little is known about shrubs at the treeline. We hypothesized that the pattern of main nonstructural carbohydrates (NSCs) across elevations depends on the interplay between phenotypic trait plasticity, plant–plant interaction, and elevation.

**Methods:**

We studied the pattern of NSCs (i.e., glucose, fructose, sucrose, and starch) in alpine stands of *Vaccinium myrtillus* (above treeline) across an elevational gradient. In the same plots, we measured key growth traits (i.e., anatomical stem features) and shrub cover, evaluating putative relationships with NSCs.

**Results:**

Glucose content was positively related with altitude, but negatively related with shrub cover. Sucrose decreased at high altitude and in older populations and increased with higher percentage of vascular tissue. Starch content increased at middle and high elevations and in stands with high shrub cover. Moreover, starch content was negatively related with the number of xylem rings and the percentage of phloem tissue, but positively correlated with the percentage of xylem tissue.

**Conclusions:**

We found that the increase in carbon reserves across elevations was uncoupled from plant growth, supporting the growth limitation hypothesis, which postulates NSCs accumulate at high elevation as a consequence of low temperature. Moreover, the response of NSC content to the environmental stress caused by elevation was buffered by phenotypic plasticity of plant traits, suggesting that, under climate warming conditions, shrub expansion due to enhanced plant growth would be pronounced in old but sparse stands.

The ability of a plant to grow and survive in different ecological conditions depends on its phenotypic plasticity (Schlichting, 1986). Among environmental gradients, the effects of elevation have been largely studied in the last decades, being considered a powerful tool to investigate the relationships between climate and vegetation (Körner, 2007). Plant acclimation to altitude has been investigated in relation to both morphological (e.g., Jonas and Geber, 1999; Weppler and Stocklin, 2004; Vitasse et al., 2010; Milla and Reich, 2011; Frei et al., 2014) and physiological traits (e.g., Smith et al., 2003; Losso et al., 2016; Pšidová et al., 2018). However, comprehensive studies on the effects of elevation on plant traits and their potential interactions are still scarce. Furthermore, many experiments focused on the response of trees and herbs, while studies on shrubs are underrepresented. For these reasons, we investigated the effect of elevation on nonstructural carbohydrates (NSCs) in underground stems of *Vaccinium myrtillus* L. (Ericaceae).

With increasing elevation, the atmospheric pressure, CO_2_ content, temperature, length of vegetation period, and nutrient availability decrease, whereas annual precipitation, frequency of frost, and solar radiation tend to increase (Körner, 2003; von Arx et al., 2006; Lütz, 2012). These conditions reduce performance and growth by generating stress in plants (Pyrke and Kirkpatrick, 1994; Körner, 2003). Exposure to low temperatures limits plant growth and reduces many physiological activities (Körner, 2003; Schulze et al., 2005; von Arx et al., 2006) producing changes on morphological (Fernández‐Calvo and Obeso, 2004; Pellissier et al., 2010) and physiological traits (Ziska et al., 1992). This might reflect on changes in ecosystem properties such biodiversity (Lomolino, 2001) and productivity (Whittaker and Niering, 1975; Raich et al., 1997).

Among plant traits, carbohydrate transport and storage have been early associated with elevation‐related stress (Mooney and Billings, 1965; Wallace and Harrison, 1978). Recently NSCs have been proposed as regulators of source‐to‐sink partitioning (Durand et al., 2018), affecting rooting of shoots and growth of roots (Schwilk and Ackerly, 2005; Palacio et al., 2007). Carbohydrate reserves enable plants to uncouple growth from C assimilation throughout the year, allowing survival under seasonal stressful conditions. Instead, soluble sugars serve as important regulators of both physiological adjustment of plants to drought and freezing stress (Larcher and Thomaser‐Thin, 1988; Meletiou‐Christou et al., 1992; Lloret et al., 2018; Yamada and Osakabe, 2018) and pathogen or herbivore attack (Tauzin and Giardina, 2014; Piper et al., 2019). Nonstructural carbohydrates are involved in frost resistance (Palacio et al., 2015; Sperling et al., 2015) by protecting plants from intracellular desiccation (Sung et al., 2003). It has been shown that NSCs are accumulated in plant tissues at the end of the growing season and kept at high levels until spring dehardening (Hagidimitriou and Roper, 1994; Larcher, 2005).

In trees, overall NSC concentrations increase with elevation, showing that temperature mainly acts on plant growth without affecting carbohydrate storage, thus supporting the growth limitation hypothesis (GLH) (Körner, 1998; Hoch et al., 2002; Fajardo et al., 2012; Hoch and Körner, 2012; Fajardo and Piper, 2014). However, plants living under similar environmental conditions may show different dynamics of carbohydrate storage and use in relation to differences in vegetative or ecological strategies (Larcher and Thomaser‐Thin, 1988; Mooney et al., 1992; Barbaroux and Bréda, 2002; Newell et al., 2002; Wang et al., 2018). The GLH has mainly been proposed for trees, whereas its validity for shrubs and perennial forbs is uncertain (Körner, 1998). Recently, in branch wood of the shrub *Myricaria elegans* a nonlinear relationship between NSCs and elevation was observed (Dolezal et al., 2019), but in smaller plants like dwarf shrubs no clear pattern of NSC in relation to elevation has been so far observed. Nevertheless, some studies have examined the effect of latitude, snow cover, and frost on resistance to drought stress, showing that NSCs are used by plants in response to environmental stress, especially in relation with snow cover (e.g., Palacio et al., 2015; Wheeler et al., 2016). For dwarf shrubs, most investigations focused on annual growth, ring widths, and shrub age, showing a solid relationship with climate variation (Rixen et al., 2004; Wipf et al., 2006; Bär et al., 2008; Myers‐Smith et al., 2015; Anadon‐Rosell et al., 2016).

Since alpine and arctic shrubs are highly sensitive to temperature and its variation (Sturm et al., 2001; Dial et al., 2007; Blok et al., 2011; Myers‐Smith et al., 2015), and the recent expansion of their communities seems to be a prominent evidence of climate change (Archer et al., 1995; Sturm et al., 2001). Therefore, the anticipated climatic warming has been hypothesized to deeply interfere with alpine heaths, by extending plant life (Shi et al., 2014), modifying plant abundance, altering nutrient content (Kaarlejärvi et al., 2012), and leading to a widespread reduction of biodiversity (Ratajczak et al., 2012; Boscutti et al., 2018). Plant–plant interactions represent one of the major selective forces driving population and community dynamics (Callaway and Walker, 1997). This effect is particularly true in shrub and tree communities under extreme conditions, such as the treeline (Grau et al., 2012). Plant–plant interactions may also interfere with carbon dynamics by affecting competition for resources (e.g., nutrients availability) and, hence, regulating plant growth (Kobe et al., 2010).

Among the arctic–alpine dwarf shrubs, *V. myrtillus* is a key species in boreal dwarf shrub communities and represents a good model to study climate‐ and environment‐driven effects at the community (Tømmervik et al., 2004; Boscutti et al., 2018) and species levels (Woodward, 1986; Martz et al., 2010). Some important contributions on the effects of elevation on *V. myrtillus* physiology have been reported (e.g., Woodward, 1986; Martz et al., 2010), whereas NSC content was studied mainly in relation to variation of geographical and seasonal allocation (Stewart and Bannister, 1973; Pakonen et al., 1991). Other studies focused on plant growth (Fernández‐Calvo and Obeso, 2004) and species interactions (e.g., Anadon‐Rosell et al., 2016).

In this work, we tested the hypothesis that phenotypic trait plasticity and plant–plant interactions are pivotal variables determining the response of alpine *V. myrtillus* stands to climate variation, here expressed by changes in elevation. In particular, we investigated the relationships between NSCs in underground reserves, elevation, growth traits (i.e., stem age and tissue area), and shrub density (i.e., shrub cover). Among carbohydrates classically detected in woody plants (Quentin et al., 2015), we analyzed the content of (1) starch, which represents the main carbohydrate reserve; (2) sucrose, used for carbon reallocation; and (3) fructose and glucose, representing the sugar pool for energetic requirements. We expected to find climate as the main driver for NSC content; therefore, we hypothesized that at higher, compared with lower elevation (i.e., low temperature), *V. myrtillus* plants may have more soluble sugars (i.e., glucose, fructose, and sucrose) and similar starch content (i.e., main NSC storage), in response to environmental stress increase and/or a limitation of the overall carbon assimilation occurs. We also hypothesized that NSCs may be related to plant traits and density. In particular, we expected all NSCs to increase in stands with higher shrub cover, due to a an increase in plant–plant interaction (i.e., more competition for nutrients) and a relative lower investment of carbon in plant growth, and in old individuals, especially when the percentage of storage tissues is higher due to a probable slower growth rate.

## MATERIALS AND METHODS

### Sites and plant communities

The study was carried out in the Fleons Valley, Carnic Alps (Friuli Venezia Giulia, Italy, 12°44′21″E, 46°38′01″N). The bedrock mainly comprises Paleozoic metamorphic siliceous sand and mudstone (Venturini, 2006). The area has a mean annual precipitation of 1170 mm, a mean annual air temperature of 3.0°C, and at 1980 m a.s.l., 197 ± 11 days have snow cover >10 cm (data provided by OSMER ARPA [Meteorological Observatory of the Regional Agency for Environmental Protection], accessed October 2019). The growing season for *V. myrtillus* stands lasts from April (lower altitude) or May (higher altitude) to September. At the study site, the most frequent species in the acidophilus dwarf‐shrub communities (*Rhododendro‐Vaccinion*) (Mucina et al., 2016) are the shrubs *Rhododendron ferrugineum* and *Vaccinium myrtillus* and the herbs *Deschampsia flexuosa*,* Arnica montana*,* Carex sempervirens*,* Homogyne alpina*, and *Solidago virgaurea* subsp. *minuta*. The area was previously influenced by livestock grazing, which ceased at least 10 years before the surveys. Grazing of wild ungulates in the area is possible, but no evidence of direct grazing was found during the field surveys.

### Sampling design

To guarantee homogenous ecological (soil, light, and microclimate) and biological (plant community) conditions, we did the study in alpine stands of *V. myrtillus* above the treeline; hence, we did not include the populations present in mountain and sub‐mountain belts, where *V. myrtillus* grows in the understory of Norway spruce forests. Sampling was conducted in 20 plots of 25 m^2^ (5 × 5 m), selected along an elevational gradient of ca. 500 m (i.e., from 1690 to 2220 m a.s.l.). This altitudinal range corresponds to a theoretical temperature difference of approximately 3°C; for this reason, a high frequency of plots (elevation interval of ca. 25 m) was set, allowing a fine tuning of the climate gradient. Plots were selected using a vegetation map and a digital elevation model (DEM) and then randomly positioned within dwarf‐shrub communities along elevation belts, using a GIS environment (ArcGIS 10.0; ESRI, Redlands, CA, USA). The plots matched the following criteria: (1) dwarf‐shrub cover >30% of the overall vegetation cover, (2) orientation between east (90°) and south (180°), and (3) slope between 20 and 30°. Site characteristics (i.e., aspect, slope) were recorded as possible covariates in the preliminary statistical models. All samples were collected in late August 2014 when fruits were ripe. At this stage, underground organs had maximal carbon reserves to face winter rest and initiation of the new growing season (Pakonen et al., 1991), representing an ideal point to trace the balance for annual carbon dynamics.

### Growth traits of *Vaccinium myrtillus* populations and plant community features

Ten underground stems with their connected aboveground structures (hereafter ramets) of *Vaccinium myrtillus* were collected in each plot, clipping them at least at 5 cm below ground level. For the dendrochronological analyses, we used the methods for herbs and shrubs proposed by Schweingruber and Poschlod (2005). All the collected stems were treated with glycerin‐alcohol and paraffin‐embedded. Cross sections of 5 μm thickness were cut from the basal 1.5 cm of each stem using a sledge microtome. The sections were stained with toluidine blue (0.1% v/v in water) to highlight the growth ring structure and then dried at 60°C for 2 h. Then, they were immersed twice for 2 min each in xylol, then in ethanol solutions (100% I, 100% II, 95%, 80%, and 50% for 1 min each), and distilled water (1 min). Images of cross sections were captured at 40–200× magnification using a Leica DMLB microscope (Leica Microsystems GmbH, Wetzlar, Germany) and a digital camera (ICC 50, Leica). Overall, 200 cross sections were analyzed. Images were used to visually count xylem rings and to measure their width in three radii per section, pooling them as average for each ramet with the plug‐in ObjectJ of the ImageJ software (Schneider et al., 2012). A subsample of three cross sections for each plot was further considered to assess xylem, phloem, and medullary ray tissue percentages. For each section, a sector of 90° was analyzed using ImageJ to measure the overall area and the area of each tissue. The percentage of each tissue for a given sector was calculated as the ratio between the area occupied by the tissue and the overall area of the sector. In each plot, the percentage of dwarf shrubs cover was estimated for specific species as well as the total cover.

### Nonstructural carbohydrates

Glucose, fructose, sucrose, and starch were considered as the most representative NSCs in woody plants (Quentin et al., 2015). Their contents were analyzed on three ramets randomly selected from the same underground samples used for growth trait analyses. Just after collection, samples 5 cm long were microwaved at 700 W for 3 min to denature oxidizing enzymes. Samples were oven‐dried at 56°C for 24 h and ground to fine powder (particle size <0.15 mm), and 15 ± 1 mg of material was transferred into a 1.5 mL Eppendorf tube and handled as proposed by Quentin et al. (2015), modified for small samples. NSCs were evaluated as NADPH formation (Bergmeyer and Bernt, 1974) with a spectrofluorimeter (LS50B Luminescence Spectrometer, Perkin‐Elmer, Waltham, MA, USA), using 330 nm excitation and 460 nm emission wavelengths. The mean values were calculated within each plot for each NSC. Bulk NSC was calculated as the sum of the analyzed carbohydrates (i.e., starch, sucrose, glucose, and fructose).

### Data analysis

The effects of elevation, plant traits, and shrub cover on NSCs were tested using linear models (LMs). Due to the high correlation between glucose and fructose (*r* = 0.91, *p* < 0.01), only the results of glucose are shown, similarly for starch and bulk NSC (*r* = 0.98, *p* < 0.01) (Appendix S1). Ring width was also discarded due to its correlation to xylem percentage (*r* = 0.8, *p* < 0.01). Each model encompassed NSCs (i.e., glucose, sucrose and starch) as dependent variables and elevation, plant growth traits (i.e., age, xylem, phloem and medullary rays), and dominant shrub cover (i.e., *V. myrtillus*,* R. ferrugineum*) as predictors. In all models, a quadratic term was also preliminarily included for each predictor to account for possible nonlinear relationships. For all models, we also preliminarily included environmental covariates, such as aspect (sine transformation, southness index) and slope. No significant effect emerged; thus, covariates were dropped from the final models. In addition, we assessed the correlation between dwarf shrub cover and the two most abundant shrub species (i.e., *V. myrtillus*:* r* = 0.5, *p* < 0.05, LM, *p* < 0.05; and *R. ferrugineum*:* r* = 0.4, *p* < 0.05, LM, *p* < 0.05). No relationship between elevation and shrubs cover was found (Boscutti et al., 2018). Model assumptions were checked by diagnostic plots of residuals. Afterward, we used multi‐model inference to evaluate the influence of variables on NSCs. We used Akaike's information criterion (AIC) to choose all the plausible models (ΔAIC < 2) (Burnham and Anderson, 2002). We also derived Akaike's model weight (wi), which represents the probability that the model is the best fitting model if the data were collected again under identical circumstances, and the relative importance of the variables (RVI), using Akaike's model weight (Burnham and Anderson, 2002). All statistical analyses were performed using the R statistical software (R Core Team, 2018). The multi‐model inference was performed using the MuMIn package (Barton, 2017).

## RESULTS

**Figure 1:**
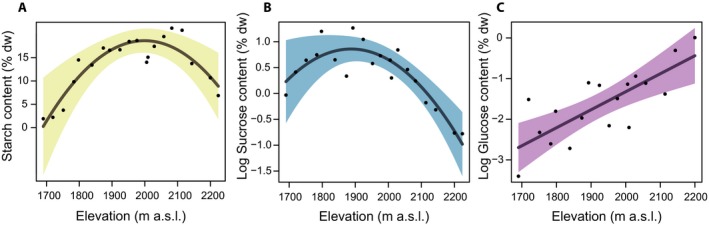
(A) Starch, (B) sucrose, and (C) glucose content in underground stems of *Vaccinium myrtillus* according to the outcomes of multi‐model inference analysis. Nonstructural carbohydrates are expressed as percentage of dry mass (% dw); sucrose and glucose contents were logarithmically transformed. Shaded area is the 95% confidence interval.

The results of multi‐model inference showed that for the starch content in the underground ramets of *V. myrtillus*, only two models were supported (Table 1, ΔAIC < 2), which explained 76% of the total variation. The content of starch, which was by far the most abundant NSC, showed a hump‐shaped pattern along elevation, increasing until ca. 2000 m a.s.l. (Fig. 1A).

**Table 1:** List of plausible models performed with multi‐model inference (ΔAIC < 2). The estimates were the intercept, the variables considered in each model (elevation = altitude [m a.s.l.]; shrub cover = abundance of shrubs as percentage of shrub cover; age = mean of number of xylem rings of ramets; xylem = percentage of ramet section occupied by xylem; phloem = percentage of ramet section occupied by phloem; medullary ray = percentage of ramet section occupied by medullary ray), their relative importance (***p < 0.001; **p < 0.01, *p < 0.05, °*p* < 0.1), *R* ^2^, df, AIC, ΔAIC, and model weight (weight).

NSC	Intercept	Elevation	*Rhododendron ferrugineum* cover	*Vaccinium myrtillus* cover	Age	Xylem	Phloem	Medullary ray	*R* ^2^	df	AIC	ΔAIC	Weight
Sucrose													
	**–4.1**	**+**			**+**	**4.75**	**10.1**		**0.76**	**8**	**15.7**	**0.00**	**0.16**
	–0.2	+			+		8.3		0.72	7	16.4	0.67	0.11
	–5.2	+		0.003	+	6.07	9.4		0.77	9	16.7	0.98	0.10
	–3.9	+	–0.002		+	4.51	9.9		0.76	9	17.5	1.77	0.06
	–4.1	+			+	4.74	9.9	0.60	0.76	9	17.7	1.97	0.06
RVI	—	1.00**	*0.33*	*0.34*	***0.87°***	*0.55*	*0.77*	*0.29*		—	—	—	—
Glucose													
	**–7.9**	**0.004**	**–0.016**	**–0.014**					**0.57**	**5**	**40.6**	**0.00**	**0.11**
	–7.7	0.004	–0.015	–0.012			–10.1		0.60	6	41.2	0.67	0.08
	–9.1	0.004	–0.011						0.49	4	41.6	1	0.07
	–8.6	0.004	–0.011				–12.8		0.54	5	41.6	1.08	0.06
	–9.0	0.004							0.42	3	41.7	1.17	0.06
	–11.1	0.004	–0.015	–0.013		2.86			0.58	6	42	1.46	0.05
	–8.5	0.004					–12.1		0.47	4	42.2	1.67	0.05
	–7.5	0.004	–0.016	–0.014				–3.53	0.57	6	42.3	1.74	0.05
	–8.3	0.004	–0.016	–0.014	0.013				0.57	6	42.5	1.98	0.04
RVI	—	0.95**	*0.63*	***0.51***	*0.32*	*0.32*	*0.41*	*0.29*		—	—	—	—
Starch													
	***–83.2***	**+**	**0.12**	**0.13**	**+**	**133.70**	**–148.1**		**0.76**	**10**	**111.2**	**0.00**	**0.52**
	*–80.0*	+	0.12	0.12	+	130.30	–141.5	–19.76	0.76	11	112.9	1.69	0.22
RVI	—	0.93**	***0.79°***	*0.77*	0.89*	***0.86°***	0.91*	*0.40*	—	—	—	—	—

**Figure 2:**
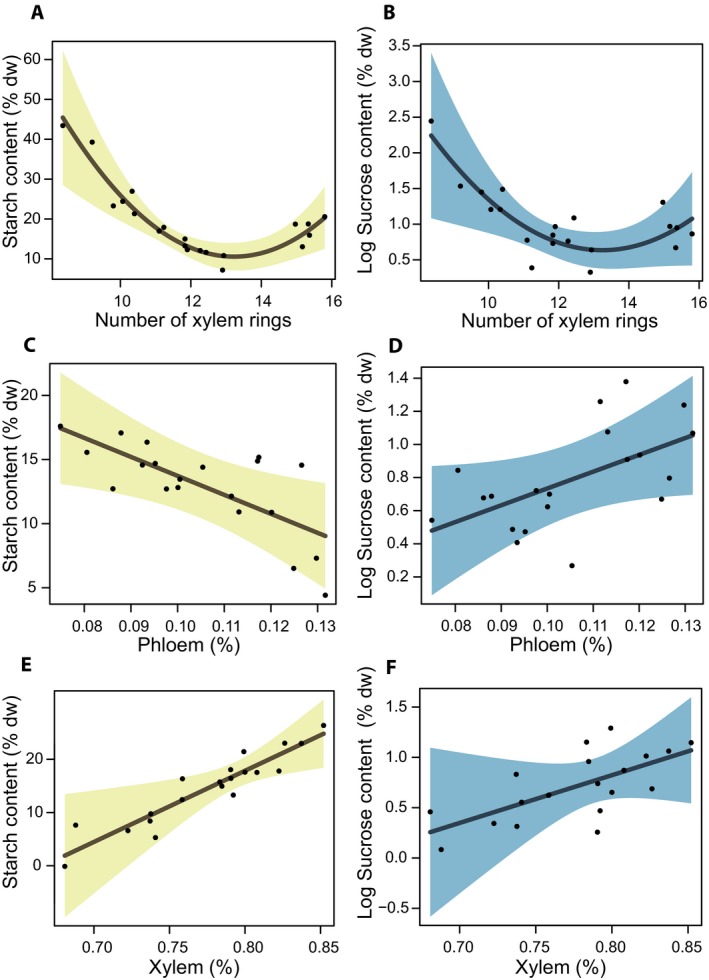
Effect plots of plant functional traits on (A, C, E) starch, and (B, D) sucrose content in underground stems of *Vaccinium myrtillus* according to the outcomes of multi‐model inference analysis. The effects of significant relationships between nonstructural carbohydrates (NSCs) and mean values of ramet sections, number of xylem rings, percentage of phloem tissue, and percentage of xylem tissue are reported. NSCs are expressed as percentage of dry mass (% dw); sucrose content was logarithmically transformed. Shaded area is the 95% confidence interval.

**Figure 3:**
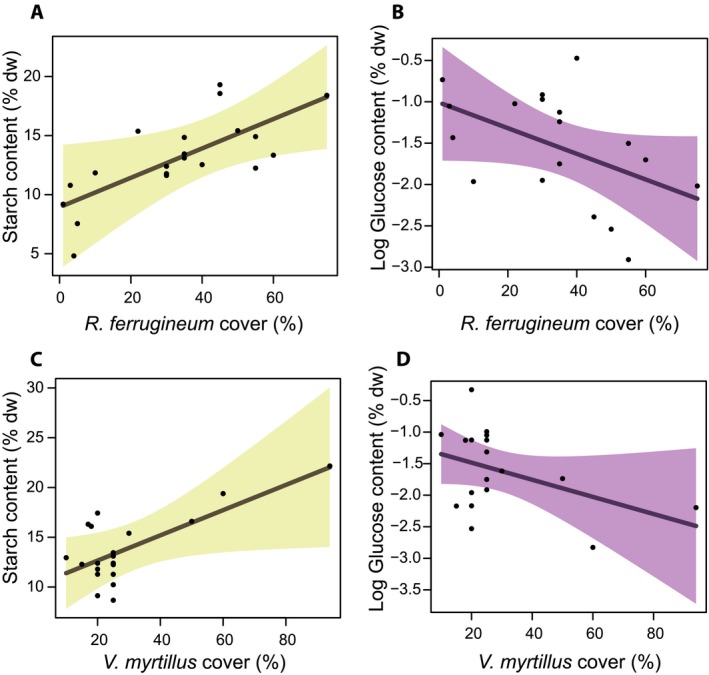
Effect plots of plant–plant interactions (i.e., mean cover of *Rhododendron ferrugineum* and *Vaccinium myrtillus*) on (A, C) starch and (B, D) glucose content in the underground stems of *V. myrtillus* according to the outcomes of multi‐model inference analysis. Nonstructural carbohydrates are expressed as percentage of dry mass (% dw); glucose content was logarithmically transformed. Confidence intervals (0.95) are also plotted.

Starch content increased in ramets with higher percentages of xylem, whereas it decreased in ramets with a high percentage of phloem and in older plants (Fig. 2A, C, E). Starch content was higher in stands with high shrub density, showing similar relationships for both *V. myrtillus* and *R. ferrugineum* (Fig. 3A–C).

The results of multi‐model inference of sucrose content showed that five models were supported, explaining ca. 75% of the total variation in sucrose content. Among the studied variables, plant traits (i.e., ramet age, phloem area, and xylem area) and elevation were present in the best model (Table 1). Sucrose content showed a hump‐shaped pattern along elevation with the highest values at mid‐elevation (ca. 1900 m a.s.l.) (Fig. 1B). Sucrose increased in ramets with higher percentages in both phloem and xylem area, whereas it decreased rapidly in ramets up to 12 years old and was undetectable in older ramets (Fig. 2B, D, F).

Finally, for glucose content, nine plausible models explained from 42 to 57% of the total variation. In the underground stems, glucose concentration (as well as fructose, due to their very high correlation; Appendix S1) was mainly affected by elevation and shrub cover. Glucose content showed a positive linear relationship with elevation (Fig. 1C), while it was negatively correlated with cover of both *V. myrtillus* and *R. ferrugineum* (Fig. 3B–D).

## DISCUSSION

Our findings explore plant responses to environmental stress, pointing out some possible links between plant acclimation, plant–plant interactions, and phenotypic plasticity, here represented by growth trait variability. We found that studying the pattern of plant carbon reserves might help to define the response of plants to climate variation in light of the growth limitation hypothesis (GLH). Moreover, these results provide new perspectives in the understanding of the ongoing expansion of tundra shrubs due to climate change.

The content of all soluble carbohydrates measured (i.e., sucrose, glucose, and fructose) was affected by elevation. Plant–plant interaction, here represented by shrub species cover, was also an important driver for starch and glucose, but not for sucrose content. Ramet age (i.e., number of xylem rings) and stem tissue area were differently related to all nonstructural carbohydrates (NSCs), showing a strong influence of the *V. myrtillus* population structure on plant carbohydrate content. These findings suggest that elevation, most likely by means of temperature, may directly influence all considered NSCs. However, we showed that plant traits of *V. myrtillus* and plant–plant interaction were pivotal in shaping NSC response along elevation. This concept underlines that plant acclimation capacity, evaluated as NSC reserves, cannot be fully interpreted without considering the interaction of functional traits and plant community.

### Relationship between elevation and NSCs

It is widely recognized that elevation affects plant growth and physiology by generating more severe environmental conditions, namely, increased exposure to low temperatures (Körner, 2003; Schulze et al., 2005; von Arx et al., 2006), and consequent reduction of the growing period also due to prolonged snow cover (Körner, 2003; Jonas et al., 2008). Nonstructural carbohydrates have been shown to respond to environmental stress associated with altitude (Fajardo et al., 2013; Dolezal et al., 2019).

We found a general increase in NSCs along elevation in *V. myrtillus* underground stems, mainly due to an increase in starch and hexoses, whereas sucrose decreased. Our findings might be interpreted as a response of *V. myrtillus* populations to the main ecological changes occurring along elevational gradients such as temperature (e.g., plant hardening and frost resistance), snow cover, and light intensity. The interactions of these factors might be responsible for the nonlinear trends observed along the studied cline, such as unexpected accumulation of starch at mid‐high elevation.

In trees, the increase in NSC content across elevations has been linked to a limitation of plant growth, as proposed by the GLH (Hoch et al., 2002; Hoch and Körner, 2012). In our study, bulk NSCs were mainly represented by starch, which was strongly affected by elevation. Starch greatly increased at mid‐high elevations where, in the same sample areas, a strong reduction of *V. myrtillus* also occurred (Boscutti et al., 2018). The NSC pattern observed in *V. myrtillus* shows the same trend already observed in Himalayan populations of *Myricaria elegans* (Dolezal et al., 2019), although *V. myrtillus* population evidenced a stronger effect of altitude. Moreover, similar to what has been described for the vegetative organs of bilberry collected at different latitudes (Pakonen et al., 1991), our results revealed that when sucrose decreased, products of its hydrolysis (fructose and glucose) increased. This condition might be due to a lack of ATP and glucose‐6‐phophate necessary to synthesize starch, as observed by Petrussa et al. (2018) in *Arum* tubers at the end of the growing season. Alternatively, the limited respiration that bilberry stands could face at high elevation (Bansal and Germino, 2010) might induce an imbalance between sucrose hydrolysis and starch synthesis. This evidence suggests that growth is uncoupled from carbon limitation across elevation also in dwarf shrubs, showing a possible link to what has been proposed for GLH in trees. Körner (1998, p. 454) stated that GLH “provides a simple explanation for the abundance of nonstructural carbohydrates and lipids as well as the high concentrations of leaf nitrogen in treeline trees and dwarf shrubs near treeline, because of a lack of “dilution by growth”. We hence propose that, similar to the treeline, the shrubline might also be “not caused by carbon shortage, but is created by sink inhibition as a result of low temperature” Körner (1998, p. 454).

*Verticillium myrtillus* may undergo tissue dehydration in the case of frost events (Tolvanen, 1997), and it was also shown that an anticipated dehardening can be the cause of winter dieback (Ogren, 1996). It is well documented that exposure of woody plants to frost, after an early start of the growing season, may induce a freeze–thaw xylem embolism (Charrier et al., 2014; Christensen‐Dalsgaard and Tyree, 2014). In this case, the availability of osmotically active sugars can help recover from hydraulic failure by vessel refilling (Nardini et al., 2011), which has been suggested to be mainly driven by accumulation and transport of soluble sugars into the empty vessels (Secchi and Zwieniecki, 2012; Trifilò et al., 2017). This mechanism could also apply to *V. myrtillus*; Ganthaler and Mayr (2015) indicated this species is highly prone to xylem embolism and refilling repair in response to drought. Our data are consistent with this evidence and would explain the rapid carbon allocation from leaves to rhizomes reported by Anadon‐Rosell et al. (2016). Other studies have suggested that the duration of snow cover also influences plant traits in *V. myrtillus*. Anadon‐Rosell et al. (2014, 2018) showed a positive relationship between soil warming (i.e. early snow melting) and plant growth and biomass, where Rixen et al. (2010) did not find any correlation with age.

Since elevation corresponds to a longer snow cover duration, we can assume that the snow cover interacts with temperature by determining xylem abundance, without modifying the age of the ramet (ring number). Concerning the influence of snow depth and duration on NSCs, a positive effect on starch concentration, and contrasting response in soluble sugars was previously found (Palacio et al., 2015; Wheeler et al., 2016; Domisch, 2017). In particular, Wheeler et al. (2016) did not find a significant interaction between elevation and snow cover. Since starch accumulation peaked around 100 m below the maximum elevation limit of growth in our study, we hypothesize the presence of an interplay between temperature and snow cover. We also found an accumulation of sucrose (5‐ to 10‐fold higher with respect to glucose) at low‐elevation stands, where the lack of snow cover might imply a higher exposure to frost stress. Here, sucrose may represent a reserve of mobile carbohydrates ready to be transferred to plant shoots in case of frost events, a key mechanism that would help explain the success of this species in such an environment.

### Effects of plant traits on NSCs

The NSC contents in the examined populations of *V. myrtillus* were differently affected by the considered plant traits, suggesting that the morphological responses of the whole population can shape the carbohydrate storage pattern along elevation. Among the traits, relative abundance of phloem and xylem was significantly correlated to sucrose content, indicating that this sugar could be used either to enhance tissue resilience to stress or to sustain the last phenological stage before the winter rest.

Glucose was not significantly correlated to phloem percentage in the stem, suggesting that sucrose might replace hexose sugars in plants that develop more phloem, and might be result from a major need to preserve active osmotic compounds in plants with low capacity for phloem transport or from interactions between vascular tissues and phloem. It has been proposed that phloem unloading could be a mechanism to contrast cavitation during the recovery of xylem conduits (Nardini et al., 2011). In general, translocation interactions may significantly affect plant drought tolerance (Sevanto, 2014), reproduction, carbon allocation, and plant relations with insects, pests, microbes, and symbionts (Plavcová et al., 2016; Savage et al., 2016). Our results confirm the pivotal role of phloem in the balance of soluble sugars.

Sucrose and starch were negatively related to the mean number of xylem rings (i.e., ramet age), with constant low values in populations older than 12–13 years. This scenario is consistent with the age limit for efficient translocation observed in *Pinus* (Zimmermann et al., 2000) and the age‐related declining growth caused by insufficient carbon availability to meet carbon demands (Martínez‐Vilalta et al., 2007; Zhang et al., 2009; Genet et al., 2010), although some findings were inconsistent (Piper and Fajardo, 2011). We found that young individuals may contain more sucrose in stem tissue, either to be ready to reallocate resources in case of sudden environmental stress or to allow a faster metabolic rate. The starch pattern was consistent with the decreasing amount of sucrose measured in older individuals, in agreement with a decreased translocating area (i.e., phloem percentage), a morphological trait strictly related to sucrose accumulation in parenchymatic tissues of underground ramets. These findings could also be explained considering that high phloem percentage can be found in plants with high reserve mobilization and high metabolic activity, i.e., due to stress responses, plant age (i.e., young individuals) or plant–plant interactions (Lintunen et al., 2016; Murcia et al., 2016; Savage et al., 2016).

### Effects of plant–plant interactions on NSCs

We hypothesize that all NSCs that we considered might also be affected by shrub abundance, here representing a proxy of plant–plant interaction intensity and competition. Indeed, we found that shrub cover of the two dominant species of the community (i.e., *V. myrtillus* and *R. ferrugineum*) significantly affected glucose and starch content of *V. myrtillus* ramets, whereas the relationship with sucrose was not significant.

Plant–plant interaction has been shown to be a key factor for carbon storage in shrubs (García‐Cervigón et al., 2013; Anadon‐Rosell et al., 2016) and, in general, in woody plants (Olano et al., 2006). In dense stands, both intra‐ and interspecific interactions can reduce metabolic performance due to higher competition for basic resources, such as light and water. For instance, summer drought may be more severe in dense stands (Bottero et al., 2017), due to root competition (Robberecht et al., 1983; Deng et al., 2006) and, hence, the synthesis of osmotically active sugars takes place at the expense of reserves. On the other hand, in *V. myrtillus* stands, high shrub cover corresponds to aerial slender and taller individuals, mainly due to more elongated annual shoots (Boscutti et al., 2018), which compete for light. Under these circumstances, also nutrient availability may be scarce due to a higher competition. When nutrients are scarce, the overall plant growth is limited, implying a higher content of storage reserves (i.e., starch) (McDonald et al., 1986; Mooney et al., 1995; Kobe et al., 2010). In fact, we found a high starch content in the underground stems of dense stands.

This fact highlights the importance of intra‐ and interspecific effects on plant metabolism to evaluate the outcome of plant–plant interactions as a function of abiotic conditions (García‐Cervigón et al., 2013). Our results shed new light on the stress gradient hypothesis (Bertness and Callaway, 1994). This hypothesis postulates that facilitative effects (or interactions) would be dominant in harsh environments, shifting to competitive ones as abiotic conditions ameliorate (Maestre et al., 2009; Soliveres et al., 2010). Considering the NSC patterns presented in our study, it is plausible to expect that the effects of shrub cover at high elevation might turn to facilitative response, but specific dedicated research is needed to validate such a hypothesis.

## CONCLUSIONS

Our results demonstrate the importance of studying a wide range of variables to understand plant responses to changes of environmental conditions. Indeed, glucose, sucrose, and starch showed different patterns with respect to environmental variables. We thus suggest that the analysis of bulk NSCs is not sufficient to depict the complex role of these compounds in responses to abiotic stresses. Furthermore, the relationships between carbohydrates, plant traits, and altitude were not linear. This result is not surprising, considering that complex systems require a systemic approach in the study between the biota and the environment (Capra and Luisi, 2014). Our findings suggest that stress may result in plastic changes of plant traits, which may buffer the negative effects of stress. On the other hand, the studied relationships between plant traits and NSCs may be evidence for an integrated stress response syndrome.

**Figure 4:**
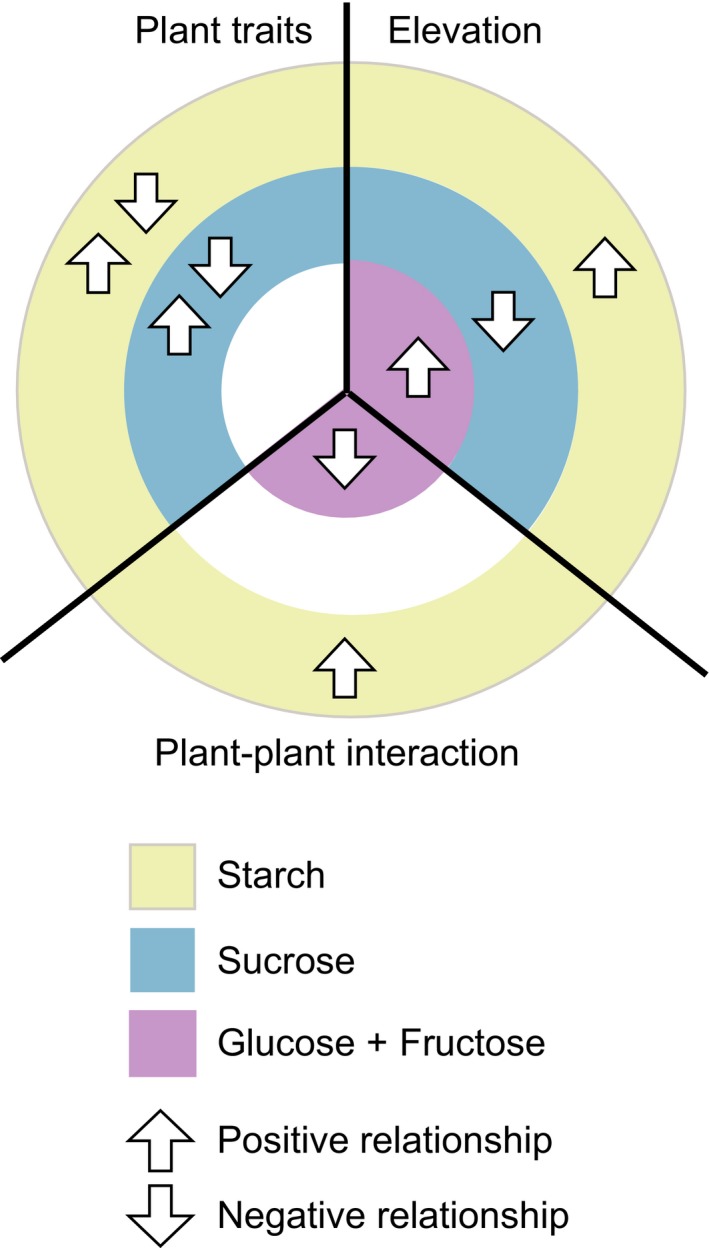
Conceptual scheme interpreting the main effects of the analyzed plant traits, plant–plant interaction, and elevation on the studied between nonstructural carbohydrates (NSCs) (starch, sucrose, glucose, and fructose) in the underground stems of *Vaccinium myrtillus*. Arrows indicate increase (upward, positive relationship) or decrease (downward, negative relationship) in content of each NSC.

As a synthesis of our findings, we propose a conceptual scheme interpreting the main effects of plant traits, plant–plant interactions and elevation on NSCs in the underground stems of *V. myrtillus* (Fig. 4). Starch was the most abundant NSC and showed significant interactions with all the predictors considered. Elevation was the main driver for all NSCs. In particular, both starch and hexoses tended to increase in stands at high elevation, consistently with what was proposed for trees in the GLH. Nonetheless, considering the general pattern of NSCs with plant–plant interactions and *V. myrtillus* population traits, some interesting trends emerged. Analyzing the coupled trends of starch and hexoses (i.e., glucose and fructose), we showed how, in addition to elevation, plant–plant interactions are also important. When plant–plant interaction was higher (i.e., high cover of *V. myrtillus* and *R. ferrugineum*), starch reached its peak, and glucose content was lower. *Vaccinium myrtillus* seems to invest glucose mainly for storage of starch when the stand has a high shrub density, while glucose is accumulated when shrub density is lower. Sucrose and starch showed coupled trends when both elevation and plant traits act together. In fact, they showed a similar pattern, except when related to phloem percentage. In the stands with ramets having a high percentage of phloem, sucrose is abundant also in relation to a low starch content. Population traits are thus the key drivers, which modulate the reciprocal content of reserves (starch) and mobile carbohydrates. In light of our findings, sucrose could be particularly sensitive to population traits and easily translocated from source to sink in response to environmental stressors. The efficiency of this mechanism is perhaps due to local morphological acclimation of the individuals, expressed by plant trait variation.

When considered at the population level, these trait changes at the individual plant level suggest that altitude results in an uncoupling between plant growth and carbon reserves, similar to the prediction for trees according to GLH. On the other hand, this phenomenon could be interpreted as a form of adaptive response (phenotypic plasticity) of the shrub community to increasing altitude. In fact, we observed nonlinear relationships between sucrose (or starch) and altitude (humped‐shape), shaped by plant traits and plant–plant interactions. Thus, NSC plasticity could be constrained by phenotypic integration, a phenomenon clearly demonstrated in plants (Valladores et al., 2007; Gianoli and Palacio‐López, 2009), also in response to altitude (Milla and Reich, 2011). Such a hypothesis deserves to be investigated, especially considering that carbon reserves are the result of a complex interplay among phenology, growth, metabolism, source‐to‐sink flow and responses to the environment (Martínez‐Vilalta et al., 2016).

This new input could be useful to understand the ongoing shrub expansion in alpine tundras, suggesting that shrub expansion due to enhanced plant growth would be pronounced in old, but sparse stands. Nonetheless, complex interactions between climate warming, ice melt and, hence, higher exposure of plants to frost stress might produce unexpected feedbacks on plant responses to climate changes. For these reasons, future studies on NSC content in alpine plants should focus on storage metabolism and on sugar allocation and transport mechanisms to shed new light on species responses to global changes.

## AUTHOR CONTRIBUTIONS

V.C.: conceptualization; data curation; investigation; visualization; funding acquisition; writing original draft. E.B.: data curation; formal analysis; project administration; validation; writing original draft; review and editing. E.P.: investigation; writing original draft; review and editing. M.Z.: investigation; writing original draft; review and editing. A.V; project administration; supervision; review and editing. F.B.: conceptualization; data curation; formal analysis; investigation; methodology; visualization; writing original draft.

## Supporting information


**APPENDIX S1.** Pearson's *r* correlation matrix between the content values of all the studied nonstructural carbohydrates (NSCs) and the bulk NSCs (expressed as sum of all considered NSCs).

## Data Availability

All data are accessible in Mendeley data repository: https://doi.org/10.17632/7md6sbmz5t.1 (Casolo et al., 2020).
