# Supplementary material for: Relationships between population traits, nonstructural carbohydrates, and elevation in alpine stands of Vaccinium myrtillus
Source: Am J Bot. 2020 Apr 1;107(4):639–49. doi: 10.1002/ajb2.1458 (PMC7217170; doi:10.1002/ajb2.1458)
Supplement: Supplementary file 1 — APPENDIX S1. Pearson's r correlation matrix between the content values of all the studied nonstructural carbohydrates (NSCs) and the bulk NSCs (expressed as sum of all considered NSCs). [file AJB2-107-639-s001.docx]

**Casolo et al.—American Journal of Botany 2020—Appendix S1**

Appendix S1. Pearson’s *r* correlation matrix between the values for all the studied nonstructural carbohydrates (NSCs) and the bulk NSCs (expressed as sum of all considered NSCs). Pearson’s *r* coefficients are reported; significant values are marked in bold *(p* < 0.001). Color gradient indicates the magnitude of negative (red) to positive (blue) correlations.

**
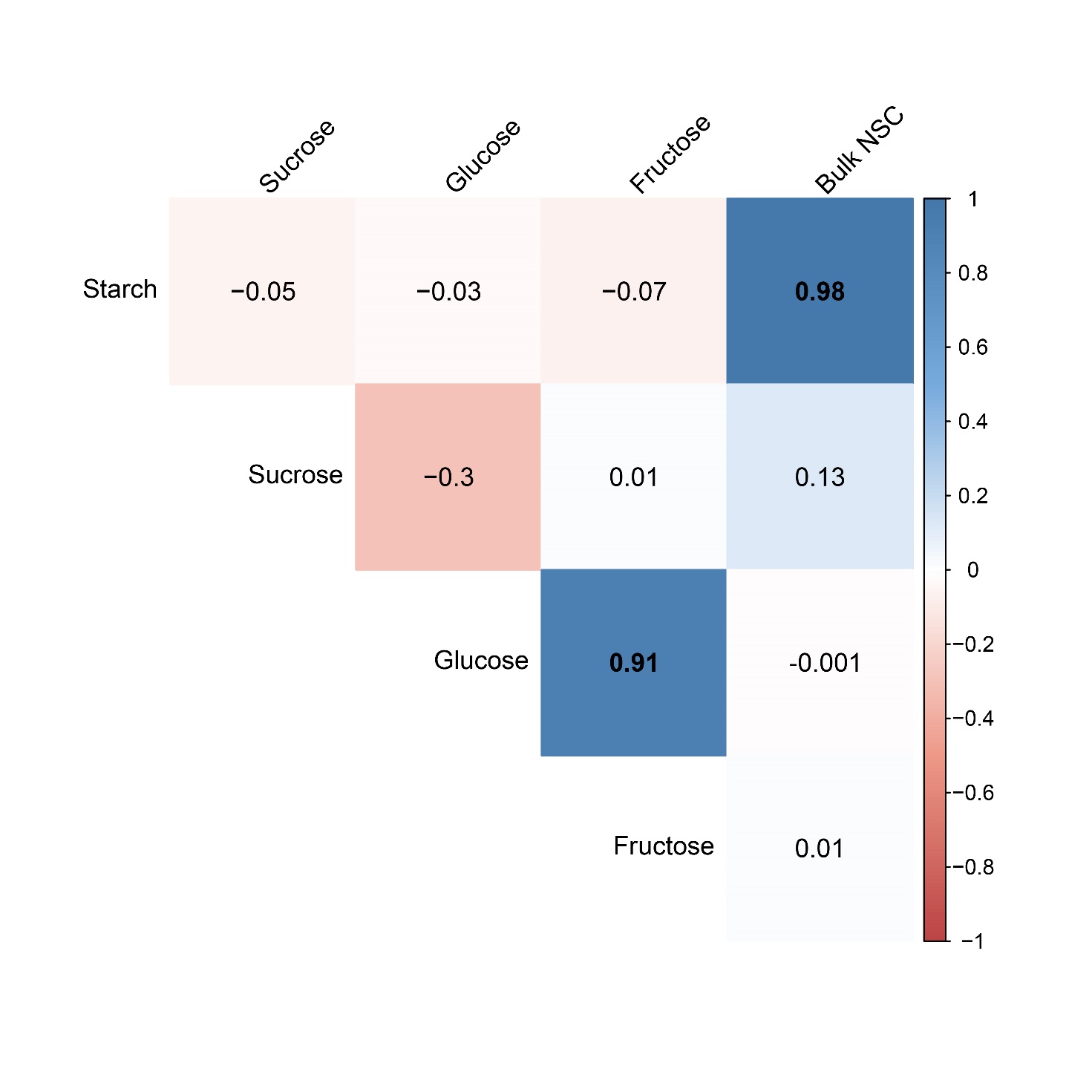
**
